# The R229Q mutation of *Rag2* does not characterize severe immunodeficiency in mice

**DOI:** 10.1038/s41598-019-39496-5

**Published:** 2019-03-14

**Authors:** Young Jin, Ara Lee, Ja Hyun Oh, Han-Woong Lee, Sang-Jun Ha

**Affiliations:** Department of Biochemistry, College of Life Science and Biotechnology, Yonsei University, Seoul, 03722 Republic of Korea

## Abstract

*RAG1* or *RAG2* mutations are associated with defects in V(D)J recombination activity, causing severe immunodeficiency with a wide spectrum of clinical phenotypes. A R229Q mutation of *RAG2* was identified in patients with severe combined immunodeficiency (SCID) or Omenn syndrome (OS). Although some factors determining the clinical features between SCID and OS were not clear, the molecular mechanism of OS was studied in a mouse model in which an EGFP tag is fused to *Rag2* with the R229Q mutation. To design the human disease model mimicking severe immunodeficiency, we generated *Rag2*-R229Q knock-in mice without an epitope tag. Mutant mice showed impaired T and B cell differentiation with reduced V(D)J recombination activity; however, the extent to which the R229Q mutation affects severe immunodeficiency was not severe. While *Rag2*-R229Q mutation under some conditions may cause severe immunological and clinical phenotypes similar to human SCID or OS, R229Q mutation per se did not cause severe immunodeficiency in mice, suggesting that additional factors other than R229Q mutation are required to induce severe immunodeficiency. Thus, our report implies that the effects of genetic background and/or a tagged protein sequence may alter the mouse immune system, revealing the mechanism of phenotypic heterogeneity arising from an identical mutation.

## Introduction

The adaptive immune response to a particular pathogen relies on B and T lymphocytes, which possess genetically rearranged and highly diverse antigen receptors^1^. During the early stage of B and T cell development, they generate a repertoire of immunoglobulins and T cell receptors by recombining variable (V), diversity (D), and joining (J) gene segments of antigen receptor loci^2^. Each of the V, D, and J gene segments is flanked by recombination signal sequences (RSSs), composed of conserved heptamer and nonamer elements which are separated by a spacer of either 12 or 23 base-pairs long^2^. The lymphocyte-specific recombination activating gene 1 (*RAG1*) and 2 (*RAG2*) initiate the process of V(D)J recombination by introducing site-specific DNA cleavage at the junction between the RSSs and the adjacent coding segment^3^. Thus, the RAG genes play an essential role in the rearrangement of the genes encoding antigen-specific receptors of B and T lymphocytes, thereby facilitating the diversity of antigen recognition present in the mature lymphocyte population^4^.

RAG deficiency in humans leads to severe combined immunodeficiency (SCID), associated with a complete absence of mature B and T lymphocytes^5^. However, patients with hypomorphic mutations in *RAG1* or *RAG2* that display partial V(D)J recombination activity can give rise to a wide spectrum of clinical and immunological phenotypes, ranging from Omenn syndrome (OS) to atypical SCID^6^. In typical OS patients, circulating B cells are mostly absent, whereas the levels of T cells are normal or elevated with a restricted T-cell receptor (TCR) repertoire^7^. Patients are defined to present atypical SCID when correlated conditions do not fully satisfy the criteria for OS^8^. While approximately 15% of the SCID-suffering infants in the United States have *RAG1* mutations^9^, the underlying molecular mechanisms of such phenotypic heterogeneity remain unclear.

Recently, crystal structure analysis of RAG protein complex revealed the role of conserved residues and frequently occurring mutations in patients^10,11^. Based on the structure of the RAG1-RAG2 protein complex, missense mutations leading to SCID or OS can be categorized into four groups: (1) mutations destabilizing the tertiary structure of RAG1-RAG2; (2) mutations affecting polar residues involved in DNA binding; (3) mutations surrounding the active sites; and (4) mutations located at the interface of RAG1 and RAG2^10^. Among these, R229 of RAG2 that forms salt bridges with D546 of RAG1^10^ is regarded to be critical for the development of SCID or OS in patients^5,8,12^. In addition, homozygous *Rag2*-R229Q/Enhanced green fluorescent protein (EGFP) mutant mice (hereafter *Rag2* KI/EGFP), developed by Marrella and colleagues^13^, presented clinical and immunological phenotypes remarkably similar to human OS with severe alopecia, erythroderma, infiltration by T lymphocytes and eosinophils into the skin and gut, and complete absence of B cells. However, this mouse model expressed the mutant protein, in which the endogenous *Rag2* gene was targeted by a construct containing *Rag2*-R229Q with an EGFP tag at the N terminus. Furthermore, they created the mutant mice by gene targeting method using mouse 129/Sv embryo-derived stem (ES) cells and performed analysis on a mixed C57BL/6 X129/Sv genetic background^13^. Although the *Rag2* KI/EGFP mice used as murine OS model helped us to understand the detailed pathogenesis of OS and autoimmunity, whether the R229Q mutation itself is sufficient to cause full development of OS and severely affect immunological disorder is still not clear.

Animal models have been providing valuable clues to the aetiology and the molecular pathogenesis of human genetic diseases caused by several types of mutations. Recently, a clustered regularly interspaced short palindromic repeat (CRISPR)/Cas9, identified in bacteria and archaea as the heritable and adaptive immune system, has become a powerful tool for genome editing in eukaryotic cells due to its accuracy, simplicity, and high efficiency^14^. The CRISPR/Cas9 system is composed of Cas9 endonuclease and a synthetic single guide RNA (sgRNA) that contains a targeting sequence (crRNA) and a Cas9 nuclease-recruiting sequence [transactivating crRNA (tracrRNA)]^14^. The sgRNA includes 20 nucleotides that are complementary to a target sequence upstream of a PAM sequence (NGG) and directs the Cas9 endonuclease to a specific location in the genome. The Cas9 endonuclease, along with an sgRNA, introduces sequence specific DNA double-strand breaks which results in non-homologous end joining (NHEJ)-mediated insertions and deletions or homology-directed repair (HDR)-mediated repair in the presence of donor templates. In mice, CRISPR/Cas9 has been successfully used for generating knockout or knock-in mice that mimic human diseases^15^.

In this study, we generated *Rag2*-R229Q knock-in mice by CRISPR/Cas9-mediated gene editing in C57BL/6 zygotes. Since *Rag2* KI/EGFP mouse model has been used to study pathogenesis of OS^13^ and the R229Q residue of *RAG2* has been considered important for lymphocyte development in patients^5,8,12^, our first intention was to set them up as a potential model for gene therapy of SCID and OS. However, our *Rag2*-R229Q mutant mouse model exhibited a milder immunological phenotype in lymphocyte development than in the previous *Rag2* KI/EGFP mouse model^13^. Furthermore, we could not observe clinical evidence for OS, possibly due to the absence of a reporter gene and/or the influence of genetic background. Accordingly, our results clearly demonstrated that identical mutation in *RAG2* give rise to distinct *in vivo* phenotypes depending on external factors, while the R229Q mutation of *Rag2* by itself is not sufficient to cause the full development of OS and SCID in mice.

## Results

### Generation of *Rag2-*R229Q mice by CRISPR/Cas9-mediated gene editing

**Figure 1:**
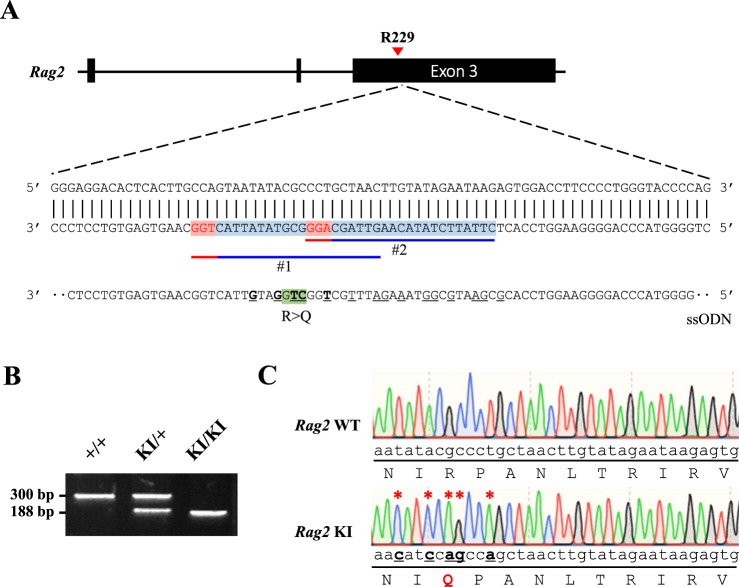
Introduction of the R229Q mutation in *Rag2* locus by CRISPR/Cas9-mediated gene editing. (**A**) A schematic representation of sgRNA targeting sites in *Rag2* and sequence of ssODN. The sgRNA targets and PAM sequences are shown in blue and red, respectively. The 173-bp ssODN donor template is presented under the genomic DNA with a R229Q mutant sequence in green. The silent mutations used to prevent re-cutting by Cas9 are underlined, whereas the bold and underlined sequences are the ssODN donor template sequences that were successfully introduced into *Rag2* locus. (**B**) PCR analysis of genomic DNA from wild-type and *Rag2*-R229Q knock-in mice. Specific bands were observed after genotyping at the expected DNA size: *Rag2* wild-type (WT) = 300 bp; *Rag2* knock-in (KI) = 188 bp. (**C**) Comparison of genomic DNA sequences between *Rag2* wild-type and *Rag2* R229Q mutation.

To establish a murine model of severe immunodeficiency with SCID or OS, we generated a *Rag2* knock-in mouse carrying the R229Q mutation by CRISPR/Cas9-mediated gene editing. The R229 residue of *RAG2*, frequently identified in patients with SCID or OS, is considered to be critical for the development of SCID or OS^5,8,12^. In accordance with this, a previous work^13^ has shown that *Rag2* KI/EGFP mice displayed immunological and clinical phenotypes similar to those of human OS; however, they generated mutant mice in which an EGFP tag is fused to endogenous *Rag2* and were bred on a mixed C57BL6 X 129/Sv genetic background. To remove the effects of genetic background and an artificial tag, multiple sgRNAs and a single-stranded oligodeoxyribonucleotide (ssODN) were designed for targeting the exon3 of *Rag2* to introduce the R229Q mutation on the pure C57BL/6 genetic background (Fig. 1A). We constructed the ssODN with silent mutations to prevent re-cleaving by CRISPR/Cas9 and to aid Polymerase chain reaction (PCR)-based genotype methods. After co-injection of Cas9 mRNA, sgRNAs, and ssODN into pure C57BL/6 zygotes, we screened the founder mice by PCR amplification on the *Rag2* locus. Among 27 newborns, we obtained one targeted knock-in mouse (Supplementary Table S1). To test germline transmission and reduce potential risk of off-target effects, the mutant mouse was continually bred with C57BL/6 wild-type mice and confirmed the presence of the *Rag2*-R229Q mutation in their progeny through genomic sequencing (Fig. 1B). The heterozygous mutant mice were subsequently intercrossed to generate homozygous mutant mice (Fig. 1C). The homozygous *Rag2*-R229Q knock-in (hereafter *Rag2* KI) mice were fertile and survived into adulthood with no obvious development defects, compared to wild-type strains (hereafter *Rag2* WT; data not shown). It is worthwhile to note that unlike *Rag2* KI/EGFP mice, our *Rag*2 KI mice did not show any pathological phenotypes of human OS such as severe alopecia and skin erythroderma (Supplementary Fig. S1).

### A mild impairment of early T cell development in *Rag2-*R229Q knock-in mice

**Figure 2:**
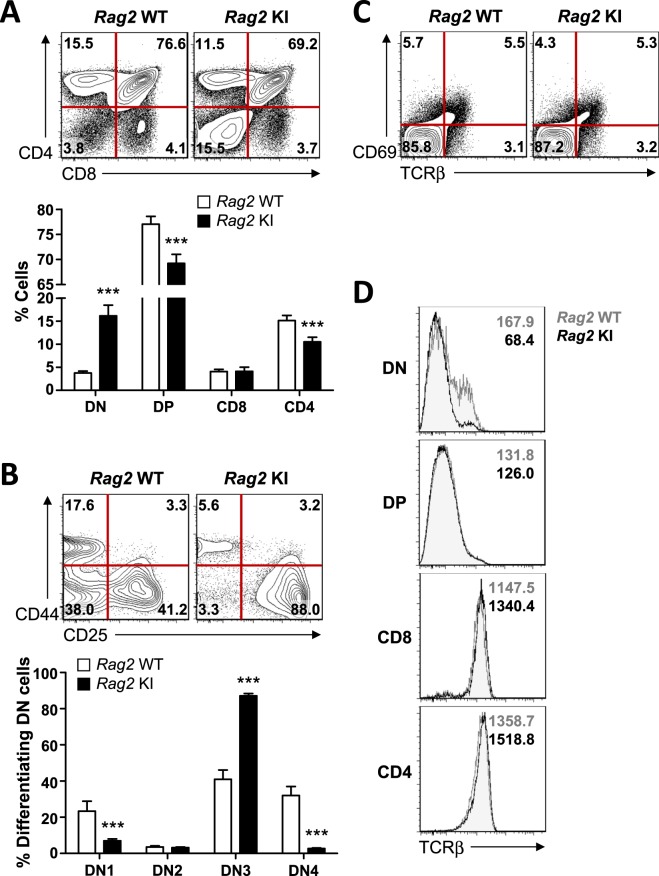
Impaired T cell development in *Rag2*-R229Q KI mice. Flow cytometric analyses were performed on wild-type (hereafter *Rag2* WT) and homozygous *Rag2*-R229Q knock-in (hereafter *Rag2* KI) mice. (**A**) Thymocytes were stained with CD8 and CD4 antibodies. Percentages of each quadrant are summarized in the bar graph (n = 8). DN, Double negative; DP, Double-positive. (**B**) CD4^−^CD8^−^ (DN) cells were gated and stained with CD25 and CD44 antibodies. Percentages of each quadrant were summarized in the bar graph. DN1, CD44^+^CD25^−^; DN2, CD44^+^CD25^+^; DN3, CD44^−^CD25^+^; DN4, CD44^−^CD25^−^. (**C**) CD4^+^CD8^+^ (DP) cells were gated and stained with TCRβ and CD69 antibodies. Numbers in the plots indicate the percentages of cells in each quadrant. Percentages of each quadrant are summarized in the bar graph. (**D**) TCRβ expressions on the indicated thymocyte subsets were analysed by flow cytometry. Gray-tinted histograms, *Rag2* WT; black histograms, *Rag2* KI. Bar graphs show mean ± standard error of the mean (SEM). n = 8 per group. ***p < 0.001.

To investigate the effects of the *Rag2*-R229Q mutation on T cell development, we performed fluorescence-activated cell sorting (FACS) analysis for thymocytes in *Rag*2 KI mice. During T cell development, immature T cell progenitors in the thymus progress from the CD4^−^CD8^−^ double-negative (DN) to the CD4^+^CD8^+^ double-positive (DP) stage. According to previous *Rag2* KI/EGFP mouse model, most thymocytes were at DN stages (3% in *Rag2* WT versus 89% in *Rag2* KI/EGFP)^13^. In contrast, only minor population of thymocytes from *Rag2* KI mice was observed at DN stage (3.8% in *Rag2* WT versus 15.5% in *Rag2* KI, Fig. 2A). Nevertheless, we found that *Rag2* KI mice showed a marked increase in the number and percentage of DN population, suggesting that thymocyte development was partially arrested at the DN stage (Fig. 2A and Supplementary Fig. S2). The DN population is divided according to the expression of CD44 and CD25, into four subsets: CD44^+^CD25^−^ (DN1), CD44^+^CD25^+^ (DN2), CD44^−^CD25^+^ (DN3), and CD44^−^CD25^−^ (DN4)^16^. TCRβ rearrangement is initiated by Dβ-Jβ recombination during transition from the DN2 to DN3 stage and completed by Vβ-DJβ joining at the DN3 stage^17^. Further analysis revealed that the DN3 cell numbers from *Rag2* KI mice were highly accumulated, whereas the DN4 cell numbers were reduced, indicating the arrest of thymocyte development at the DN3 stage (Fig. 2B and Supplementary Fig. S2). While mice lacking either RAG1 or RAG2 displayed a complete block in thymocyte development at the DN3 stage^18,19^, our data support that the R229 residue of *Rag2* affects rearrangement of TCRβ locus.

To further characterize the population of DP cells, we analysed the cells using antibodies to TCRβ and CD69. Despite the arrest of thymocyte development at the DN3 stage, the percentage of DP cells undergoing positive selection was comparable in both *Rag2* WT and KI mice (5.5% in WT versus 5.3% in KI, Fig. 2C). While positively selected thymocytes are developed into mature CD4^+^ and CD8^+^ single-positive (SP) T cells that express TCRαβ, CD4^+^ and CD8^+^ SP T cells from the thymus of *Rag2* KI mice exhibited high TCRβ expression in both *Rag2* WT and KI mice, suggesting that surviving T cells were accumulated as SP T cells (Fig. 2D). Taken together, our results indicate that *Rag2* KI mice exhibited enhanced accumulation of thymocytes at the DN stage and led to the impairment of thymocyte development at the DN-to-DP transition. However, the majority of surviving T cells were typically differentiated to CD4^+^ and CD8^+^ SP T cells in the thymus of *Rag2* KI mice.

### An alteration of T cell differentiation in the periphery of *Rag2-*R229Q knock-in mice

**Figure 3:**
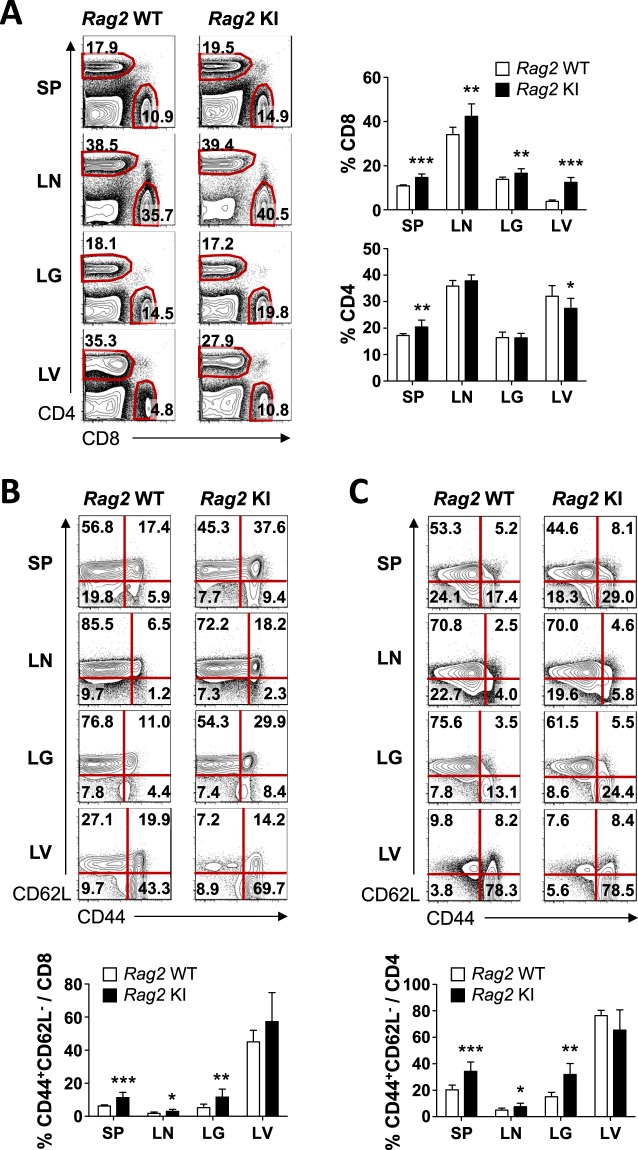
T cells and their phenotype in lymphoid and non-lymphoid organs. Cells were isolated from the spleen (SP), lymph node (LN), lungs (LG), and liver (LV) of *Rag2* WT and KI mice. (**A**) Expressions of CD8 and CD4 on lymphocytes were analysed by flow cytometry. Numbers in the plots indicate the frequency of CD8^+^ and CD4^+^ cells in the indicated tissues, and the frequency of CD8^+^ and CD4^+^ cells were summarized in the bar graph. (**B**,**C**) CD8^+^ (B) and CD4^+^ (**C**) T cells were analysed for CD44 and CD62L expression. Numbers in the plots indicate the percentages of cells in each quadrant. Frequency of CD44^+^CD62L^−^ are summarized in the bar graph. Bar graphs show mean ± SEM. n = 8 per group. *p < 0.05, **p < 0.01, ***p < 0.001.

We then examined the T cell compartment in peripheral lymphoid organs (spleen and inguinal lymph nodes) and non-lymphoid organs (lung and liver). Consistent with the reduction in the number of T lymphocytes in the thymus, the absolute numbers of lymphocytes in peripheral lymphoid and non-lymphoid organs were mostly reduced in *Rag2* KI (Supplementary Fig. S3). However, the percentages of CD8^+^ as well as CD4^+^ T lymphocytes were varied between tissues, suggesting the abnormal distribution of peripheral T cells (Fig. 3A). SP T cells in the thymus can be divided into naïve and memory cells by the expression of CD44 and CD62L^20^. In patients with OS, it has been reported that T cells are highly activated in the peripheral blood^21^. Moreover, *Rag2* KI/EGFP mice showed that activated T cells infiltrate gut and skin and the majority (~80%) of T cell subsets from lymph nodes were CD44^+^CD62L^−^, implying that SP T lymphocytes were predominantly present as effector/memory-like cells^13^. However, the percentage of CD44^+^CD62L^−^ T cells from lymph nodes of *Rag2* KI mice was slightly increased (Fig. 3B,C). Therefore, these results suggested that although the Rag2-R229Q mutation affects T cell differentiation in periphery, the extent to which the mutation contributes to T cell activation was not as severe as previously reported from *Rag2* KI/EGFP mice^13^.

### Enhanced effector/memory-like phenotype of FoxP3^+^CD4^+^ regulatory T cells in *Rag2-*R229Q knock-in mice

**Figure 4:**
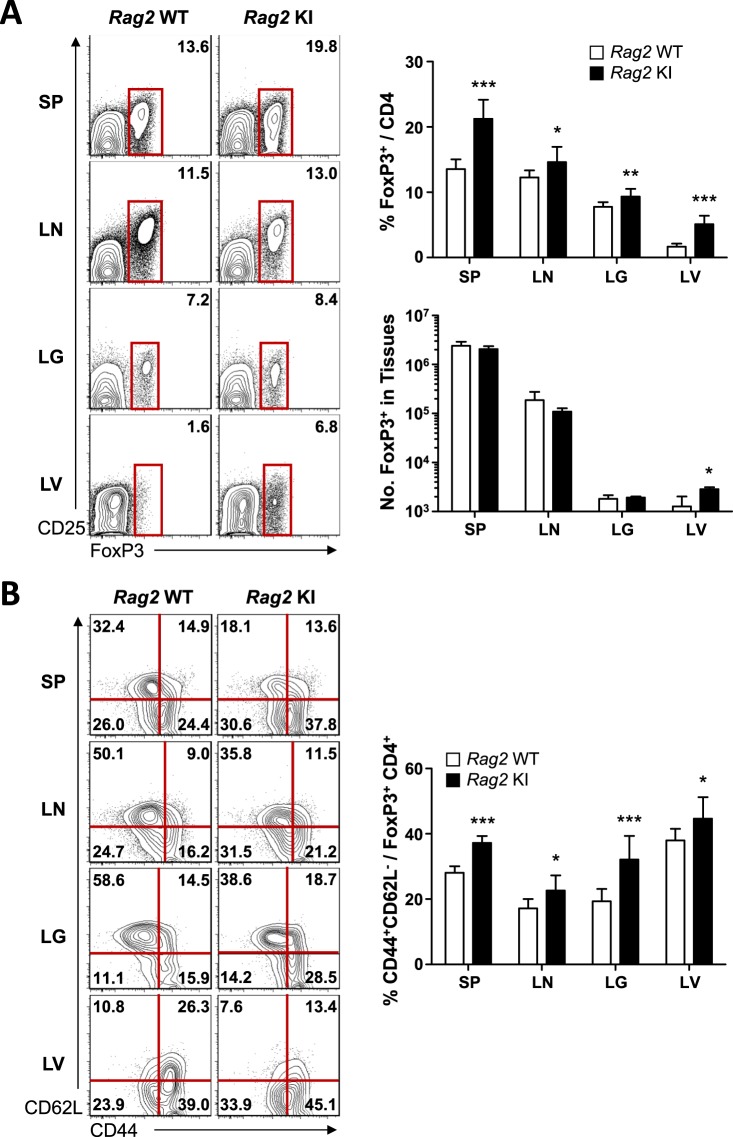
FoxP3^+^ Treg cells in *Rag2* KI mice. Cells were isolated from the spleen (SP), lymph node (LN), lungs (LG), and liver (LV) of *Rag2* WT and KI mice. (**A**) Expressions of FoxP3 and CD25 on CD4^+^ T cells were analysed by flow cytometry. Numbers in the plots indicate the frequency of FoxP3^+^ cells in the indicated tissues, and the frequency and absolute number of FoxP3^+^ cells are summarized in the bar graph. (**B**) FoxP3^+^ Treg cells were analysed for CD44 and CD62L expression. Numbers in the plots indicate the percentages of cells in each quadrant. Frequency of CD44^+^CD62L^−^ cells is summarized in the bar graph. Bar graphs show mean ± SEM. n = 8 per group. *p < 0.05, **p < 0.01, ***p < 0.001.

Next, we determined the presence of naturally occurring FoxP3^+^CD4^+^ regulatory T (Treg) cells, which play a crucial regulatory role in the pathogenesis of autoimmune diseases^22^. Interestingly, OS patients generally develop autoimmune phenomena with immunodeficiency^23^. However, a possible explanation for this paradoxical observation is the severe reduction in the number of Treg cells in *Rag2* KI/EGFP mice^13^. To test whether Treg cell population was changed or not in *Rag2* KI mice, we analysed the expression of FoxP3 that acts as the transcription factor of regulatory T cell lineage specification on CD4^+^ T cells. Surprisingly, FACS analysis revealed that the percentage of CD4^+^ T cells expressing FoxP3 was increased in *Rag2* KI mice, while the absolute numbers of FoxP3^+^ Treg cells were comparable between *Rag2* WT and KI mice (Fig. 4A). Moreover, FoxP3^+^CD4^+^ Treg cells obtained from the spleen, inguinal lymph nodes, lungs, and livers displayed enhanced effector/memory-like (CD44^+^CD62L^−^) phenotype in *Rag2* KI mice (Fig. 4B). Therefore, these findings indicate that the R229Q mutation of *Rag2* per se does not contribute to the reduction of Treg cells and the development of autoimmunity.

### Little disparity of TCRVβ diversity in *Rag2-*R229Q knock-in mice

**Figure 5:**
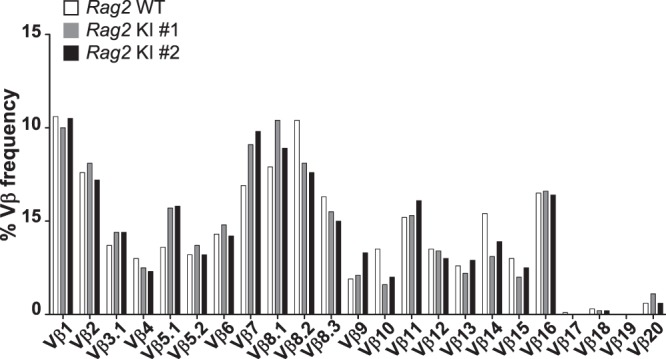
TCR repertoire of T cells in the spleen of *Rag2* KI mice. The frequency of TCRVβ repertoire was determined by next generation sequencing (NGS) analysis of T cells from one spleen of *Rag2* WT and two spleens of *Rag2* KI mice.

Since T cell development of *Rag2* KI mice was arrested at the DN3 stage, we further investigated the repertoire of T cell receptor beta chain variable genes (TCRVβ) from the spleens of *Rag2* WT and KI mice. As shown in Fig. 5, there was no significant difference in the frequencies of TCRVβ family members between *Rag2* WT and KI mice. We also analysed the TCRVβ diversity by diverse indices such as Shannon-Weaver index H, Inverse Simpson’s index 1/λ, Pielou’s evenness, and Diversity Evenness score (DE50). Although the diverse indices proposed that there was a decrease in the scores representing the TCRVβ diversity in *Rag2* KI mice, the difference between *Rag2* WT and KI mice was not statistically significant (Table 1).

**Table 1:** TCRVβ repertoire diversity indices.

Diversity Index	Normal range	Normal mean	*Rag2* WT	*Rag2* KI #1	*Rag2* KI #2
Shannon-Weaver index H'	8.922–10.283	9.56	10.59	9.88	10.21
Inverse Simpson’s index 1/λ	5191–22436	11418	19039	3070	5754
Pielou’s evenness	0.926–0.992	0.958	0.964	0.912	0.94
DE50	0.121–0.394	0.195	0.197	0.104	0.153

Diversity of repertoire is described by Shannon-Weaver index H’, Inverse Simpson’s index 1/λ, Pielou’s evenness, and Diversity Evenness score (DE50). For each index, greater score indicates higher diversity.

### A mild impairment of B cell development in *Rag2-*R229Q knock-in mice

**Figure 6:**
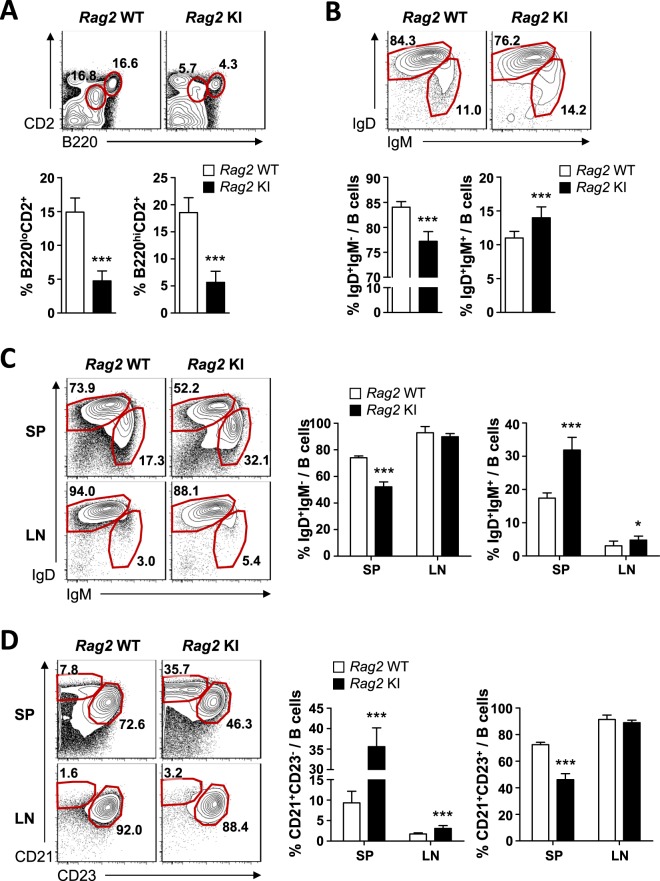
Impaired B cell development in *Rag2* KI mice. Cells were isolated from the bone marrow (BM) (**A** and **B**), spleen (SP), and lymph node (LN) (**C** and **D**) of *Rag2* WT and KI mice. (**A**) Expressions of B220 and CD2 on lymphocytes were analysed by flow cytometry. Numbers in the plots indicate the percentage of B220^10^CD2^+^ and B220^hi^CD2^−^ cells in BM. The frequency of B220^lo^CD2^+^ or B220^hi^CD2^−^ cells is summarized in the bar graph. (**B**) Expressions of IgM and IgD on B220^+^ cells were analysed by flow cytometry. Numbers in the plots indicate the frequency of IgD^+^IgM^−^ and IgD^+^IgM^+^ cells in BM. The frequency of IgD^+^IgM^−^ and IgD^+^IgM^+^ cells is summarized in the bar graph. (**C**) Expressions of IgM and IgD on B220^+^ cells were analysed by flow cytometry. Numbers in the plots indicate the frequency of IgD^+^IgM^−^ and IgD^+^IgM^+^ cells in SP and LN. The frequency of IgD^+^IgM^−^ or IgD^+^IgM^+^ cells is summarized in the bar graph. (**D**) B220^+^ cells were analysed for CD21 and CD23 expression. Numbers in the plots indicate the percentages of CD21^+^CD23^−^ and CD21^+^CD23^+^ cells in SP and LN. Frequency of CD21^+^CD23^−^ or CD21^+^CD23^+^ cells is summarized in the bar graph. Bar graphs show mean ± SEM. n = 8 per group. *p < 0.05, ***p < 0.001.

In patients with OS or SCID, circulating B lymphocytes are generally absent^23^. We therefore accessed the role of Rag2-R229Q mutation in B cell development. Although the frequency and absolute number of B220^+^ cells from *Rag2* KI mice were reduced in the bone marrow, spleen, and inguinal lymph nodes compared with *Rag2* WT mice, B cells were not absent (Supplementary Fig. S4). To delineate the transition of B cells, we stained bone marrow cells with B220 and CD2 antibodies. *Rag2* KI mice showed a significant reduction in B220^10^CD2^+^ pre-B cells and B220^hi^CD2^+^ cells including immature and mature recirculating B cells (Fig. 6A and Supplementary Fig. S4). The immature transitional B cells migrate to the peripheral blood and complete their maturation in the spleen^24^. To discriminate between transitional B cells and mature recirculating B cells, we further discriminated them by the expression of IgM and IgD. FACS analyses from bone marrow cells revealed that the percentage of IgD^+^IgM^−^ transitional B cells was reduced in *Rag2* KI mice, while the absolute numbers of IgD^+^IgM^−^ transitional B cells were comparable between *Rag2* WT and KI mice (Fig. 6B and Supplementary Fig. S4). In contrast to transitional B cells, the percentage of mature recirculating B cells was increased in *Rag2* KI mice (Fig. 6B). However, the number of mature recirculating B cells was decreased, possibly due to a reduction in total B cells from the bone marrow of *Rag2* KI mice (Supplementary Fig. S4). We next examined the percentages of IgD^+^IgM^−^ transitional B and IgD^+^IgM^+^ mature follicular B cells in the spleen and lymph nodes. While the percentage of IgD^+^IgM^−^ transitional B cells was only reduced in the spleen of *Rag2* KI mice, that of IgD^+^IgM^+^ mature follicular B cells was increased in both the spleen and lymph nodes of *Rag2* KI mice (Fig. 6C). Further analysis was performed with CD21 and CD23 antibodies to discriminate between mature follicular B and marginal zone B cells. The frequency of CD21^+^CD23^+^ mature follicular B cells was increased whereas that of CD21^+^CD23^−^ marginal zone B cells was decreased (Fig. 6D). However, absolute numbers of transitional, mature follicular, and marginal zone B cells were reduced in peripheral lymphoid organs of *Rag2* KI mice compared with *Rag2* WT mice except those of marginal zone B cells in the spleen (Supplementary Fig. S4). Accordingly, these results indicate that *Rag2*-R229Q mutation impairs B as well as T cell development; however, the effect is not as severe as previously reported from *Rag2* KI/EGFP mutant mice^13^.

**Figure 7:**
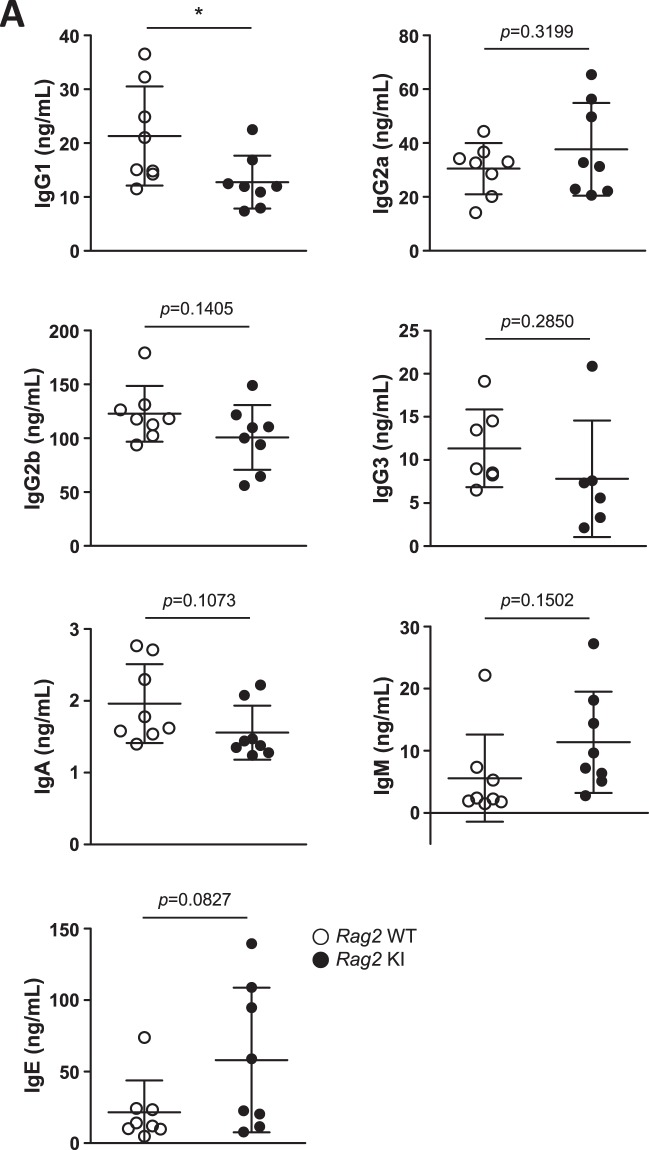
Serum concentration of immunoglobulins. Sera were collected from *Rag2* WT and KI mice. Their IgG1, IgG2a, IgG2b, IgG3, IgA, and IgM were evaluated by LegendPlex analysis, and their IgE production was measured by ELISA. Lines show mean ± SEM. n = 8 per group. *p < 0.05.

OS patients are generally characterized by alopecia, skin erythroderma, and severe immunodeficiency. As mentioned above, we could not observe any symptoms related to alopecia and erythroderma with age in *Rag2* KI mice (Supplementary Fig. S1). Besides, *Rag2* KI mice did not lead to a severe immunodeficiency with T^−^B^−^ SCID or T^+^B^−^ OS. Additional OS phenotypes present low serum immunoglobulins but paradoxically, elevated serum IgE. However, there were no significant changes in serum immunoglobulins except IgG1 (Fig. 7). Taken together, the results presented here suggest that R229Q mutation per se does not lead to severe immunodeficiency in mice.

## Discussion

The relationship between genotype and phenotype in monogenic diseases is important to predict the onset, penetrance, and severity of diseases. However, accumulating evidence suggests that identical or similar RAG mutations in relatives can lead to different clinical phenotypes^12,25–27^, indicating that mutations involved in V(D)J recombinase activity are not solely responsible for the clinical outcome. Therefore, investigating the pathogenesis of the RAG gene variant is a challenging task. Although, several algorithms have been developed to predict the pathogenicity of the RAG gene variants, these approaches have failed to elucidate the cause of the phenotypic variation^23^. While several studies proposed that additional factors may be required for determining distinct phenotypes from identical or similar RAG mutations^8^, there was no direct evidence that such factors are important contributors to the development of different phenotypes in mice. In the present study, we generated the *Rag2* KI mice by the CRISPR/Cas9 system and compared the immunological phenotypes with *Rag2* KI/EGFP mice as previously reported^13^. Intriguingly, *Rag2* KI mice displayed milder immunological phenotypes than *Rag2* KI/EGFP mice and there were no clues to the determinants of OS phenotypes including severe alopecia, B cell numbers, and elevated IgE levels in *Rag2* KI mice. Therefore, R229Q mutation per se is not sufficient for the development of OS and SCID in mice.

The R229Q mutation of *Rag2* is well described in patients leading to severe immunodeficiency with T^−^B^−^SCID or OS^5,8,12^. Biochemical analysis of recombinant RAG2 proteins suggests that the R229 residue is critical for DNA binding and catalysis^28^. While *Rag2* KI/EGFP mice recapitulate human OS^13^, we initially expected that our *Rag2* KI mice will give rise to an immunodeficiency characterized by SCID or OS. How can identical R229Q mutations give rise to different immunological phenotypes in mice? The discrepancy may be explained by the existence of additional factors required for the development of OS. One possibility is the potential effect of EGFP on *Rag2*-R229Q mutation. Although the EGFP-tagged gene served as a useful reporter system to monitor its tissue specific gene expression in living animals, growing evidences suggested that EGFP itself can alter important cellular functions such as protein stability^29^. In RAG2-transgenic mice, it was revealed that the half-life of RAG2-GFP was 2–3 days, probably much longer than that of the endogenous RAG2 protein^30^. Since the expression of RAG proteins is restricted to immature lymphocytes, the long-term effect of R229Q mutation on lymphocytes of *Rag2* KI/EGFP mice can lead to severe immunodeficiency. Another plausible explanation is the influence of genetic background. We established the *Rag2* KI mice on a C57BL/6 background, while all experiments from Rag2 KI/EGFP were performed on a mixed 129/Sv and C57BL/6 genetic background. However, we previously reported that the *Rag2* knockout mice, generated by CRISPR/Cas9-mediated gene editing on C57BL/6 and FVB/N genetic backgrounds, showed similar immunological phenotypes observed on the mixed 129/Sv and C57BL/6 genetic background^31^. Based on such observations, we thought that the EGFP tag might be a major cause of the discrepant phenotypes of *Rag2*-R229Q knock-in mice. Nevertheless, we may not exclude the potential effects of the genetic background in the mice. Therefore, our results suggest that phenotype variability in a given genotype may stem from the difference in the gene constructs and mouse strains.

Since the RAG proteins are not expressed in mature lymphocytes, the steps required for isolation of immature lymphocytes from patients should be preceded by the appropriate method. For this reason, it was difficult to study the pathogenesis of the RAG variants *in vivo*^23^. Therefore, the development of mouse models mimicking human immunodeficiency (SCID and OS) allowed us to investigate the pathogenesis of immunological disorders. Although our data suggested that an identical mutation in *RAG2* can give rise to distinct phenotypic patterns with heterogeneous clinical manifestations, there is little debate about the importance of mouse models. In parallel, hypomorphic RAG mutations were recently identified in patients with combined immunodeficiency associated with granulomas and/or autoimmunity (CID-G/AI)^32^. However, the lack of appropriate mouse models recapitulating the CID–G/AI is a stumbling block to characterize the pathophysiology of this phenotype^23^. Overall, our data propose that generating mouse models for heterogeneous conditions should consider the pathogenic mutations and mouse strains with the complexity of factors affecting the *in vivo* phenotypes.

## Materials and Methods

### Preparation of CRISPR/Cas9 mRNA and sgRNAs

Cas9 mRNA was synthesized as described previously^33^ with slight modifications. In brief, we used the mMESSAGE mMACHINE T7 Ultra kit (ThermoFisher Scientific, #AM1345) to synthesize Cas9 mRNA. The resulting products were diluted into diethyl pyrocarbonate (Sigma Aldrich, #D5758)-treated injection buffer (0.25 mM EDTA pH 8.0 and 10 mM Tris pH 7.4). The sgRNAs targeting exon3 of *Rag2* were obtained from ToolGen, Inc. (Seoul, Republic of Korea). To avoid potential risk of off-target effects, each sgRNA was designed to recognize a specific target sequence that does not share homology with any sequence of mouse genome. After screening the sgRNA cleavage efficiency *in vitro*, two sgRNAs were selected for zygote injection. The sequences of *Rag2* sgRNAs used in injection are listed as follows: sgRNA #1 (5′- GTTAGCAGGGCGTATATTACTGG-3′) and sgRNA #2 (5′-CTTATTCTATACAAGTTAGCAGG-3′).

### Preparation of the donor template

The ssODN donor template was designed to contain 173 nucleotides with homology arms, a R229Q mutation at *Rag2* gene, and blocking silent mutations to prevent re-cutting by Cas9 to aid PCR-based genotype analysis. Polyacrylamide gel electrophoresis (PAGE)-purified ssODN, oriented in the antisense direction, was synthesized from Integrated DNA Technologies (IDT, USA) and dissolved in the injection buffer (0.25 mM EDTA pH 8.0 and 10 mM Tris pH 7.4). The sequence of the ssODN was as follows: 5′-CACTGGAGACAGAGATTCCTCCTGGCAAGACTGTGCAATTCACTGCTGGGGTACCCAGGGGAAGGTCCACGCGAATGCGGTAAAGATTTGCTGGCTGGATGTTACTGGCAAGTGAGTGTCCTCCCAAAATATAAACGGTATCGTTTCTGGCAATAGAAACATGAAAAGACAGC-3′

### Animals and microinjection

C57BL/6JBomTac mice were purchased from Taconic Biosciences (Dae Han Biolink Co., Ltd., Chungbuk, Republic of Korea). To obtain mouse zygotes, female C57BL/6J mice (6‒8 weeks) were superovulated by intra-peritoneal injections of 5 IU pregnant mare serum gonadoptropin (Daesung Inc., Republic of Korea) and 5 IU human chorionic gonadotropin (Daesung Inc., Republic of Korea) at a 48-h interval. Mouse zygotes were collected by mating the superovulated females and wild-type C57BL/6J males. Prior to microinjection, Cas9 mRNA (20 ng/μl), two *Rag2*-sgRNAs (100 ng/μl in each), ssODN (200 ng/μl), and Scr7 (1 mM) were mixed in injection buffer (0.25 mM EDTA, pH 8.0 and 10 mM Tris, pH 7.4) and injected into the cytoplasm using a piezo-driven manipulator (Prime Tech). The manipulated embryos were transferred into the oviducts of foster mothers to produce mutant mice. All animal care and experiments were performed in accordance with the guidelines for Korean Food and Drug Administration (KFDA) and were approved by the Institutional Animal Care and Use Committees (IACUC) of the Laboratory Animal Research Center at Yonsei University (Permit Number: 201507-390-01). All mice were maintained in the specific pathogen–free (SPF) facility of the Yonsei Laboratory Animal Research Center.

### Founder screening and genotyping PCR

Founder mice were identified by PAGE-based PCR genotyping from tail biopsies^34^. PCR amplification was performed with the primers encompassing the sgRNA target sites. Following PAGE-based PCR genotyping, the resulting PCR products were isolated using the DNA purification kit (iNtRON Biotechnology, #17290) and then cloned into the TA cloning vector (SolGent Co., Ltd., Republic of Korea). The mutant sequence of cloned PCR products was confirmed by DNA sequencing. For routine genotyping, the following primers were used for detecting both wild-type and mutant alleles: WT-F (5′-TGT CCCTGAACCCAGATACG-3′), MT-F (5′-AACATCCAGCCAGCT-3′), WT-R (5′-TTAGCAGGGCGTATATTACT-3′), and MT-R (5′-AGGCTGCAGACCATCCTTTT-3′).

### Sample processing for the flow cytometry analysis

To obtain single-cell suspensions, lymphocytes were isolated from the thymus, spleen, inguinal lymph nodes, lungs, and livers as described before^35^. The prepared cells were stained with the following antibodies: anti-CD4-BV605 (RM4–5, BioLegend), anti-PD-1-BV421 (RMP1–13, BioLegend), anti-CD2-PE-Cy7 (RM2–5, BioLegend), anti-CD23-PerCP-Cy5.5 (B3B4, BioLegend), anti-CD21/35-BV421 (7E9, BioLegend), anti-CD43-PE (1B11, BioLegend), anti-IgM-APC (RMM-1, BioLegend), anti-IgD-BV605 (11-26c.2a, BioLegend), anti-CD8-PerCP-Cy5.5 (53-6.7, eBioscience), anti-CD25-FITC (PC61.5, eBioscience), anti-CD3-PE-Cy7 (145-2C11, BD Biosciences), anti-CD44-V450 (IM7, BD Biosciences), anti-CD45R/B220-FITC (RA3-6B2, BD Biosciences), anti-CD62L-FITC (MEL-14, BD Biosciences), anti-CD69-PE (H1.2F3, BD Biosciences), and anti-TCR-β-FITC (H57-597, BD Biosciences). For the staining of transcription factor FoxP3, the cells were stained using anti-FoxP3-APC (FJK-16s, eBioscience) after permeabilization/fixation step. In all experiments, cells were labelled with the Live/Dead fixable Stain Kit (eBioscience) to discriminate the live and dead cells. The data were acquired by FACS CANTO II (BD Biosciences) and analysed by FlowJo software (Tree Star).

### Analysis of serum immunoglobulins

Serum immunoglobulin levels (IgG1, IgG2a, IgG2b, IgG3, IgA, and IgM) of mice were analysed by a multiplex bead-based assay panel (BioLegend, cat #740495) using fluorescence-encoded beads. The samples were prepared according to manufacturer’s instructions and analysed by FACS CANTO II (BD Biosciences). The serum immunoglobulin level of IgE was measured using a Mouse IgE ELISA MAX kit (BioLegend).

### TCRVβ repertoire analysis

CD90.2^+^ cells were isolated from the spleen using positive selection (Miltenyi Biotec). The isolated cells were stored in RNAprotect® cell reagent (QIAGEN), and quantitative TCRVβ repertoire was determined by Repertoire Genesis, Inc. based on next generation sequencing (NGS) analysis. The diversity of TCRVβ repertoire analysis is described by Shannon-Weaver index H’, Inverse Simpson’s index 1/λ, Pielou’s evenness, and Diversity Evenness score (DE50), which are provided by Repertoire Genesis, Inc.

### Statistical analysis

Data were analysed by two-tailed unpaired Student’s *t*-test using GraphPad Prism software. A *p* value less than 0.05 was considered statistically significant.

## Supplementary information

Supplementary Information

## Data Availability

The datasets generated during and/or analysed during the current study are available from the corresponding author on reasonable request.
