# Supplementary material for: The R229Q mutation of Rag2 does not characterize severe immunodeficiency in mice
Source: Sci Rep. 2019 Mar 14;9:4415. doi: 10.1038/s41598-019-39496-5 (PMC6418226; doi:10.1038/s41598-019-39496-5)
Supplement: Supplementary file 1 — Supplementary Information [file 41598_2019_39496_MOESM1_ESM.pdf]

The R229Q mutation of *Rag2* does not characterize severe immunodeficiency in mice

Young Jin[#], Ara Lee[#], Ja Hyun Oh, Han-Woong Lee[*], and Sang-Jun Ha[*]

Department of Biochemistry, College of Life Science and Biotechnology, Yonsei University, Seoul 03722, Republic of Korea

[#]-These authors contributed equally to this paper

[*]-Correspondence to Han-Woong Lee (e-mail: hwl@yonsei.ac.kr) or Sang-Jun Ha (e-mail: sjha@yonsei.ac.kr)

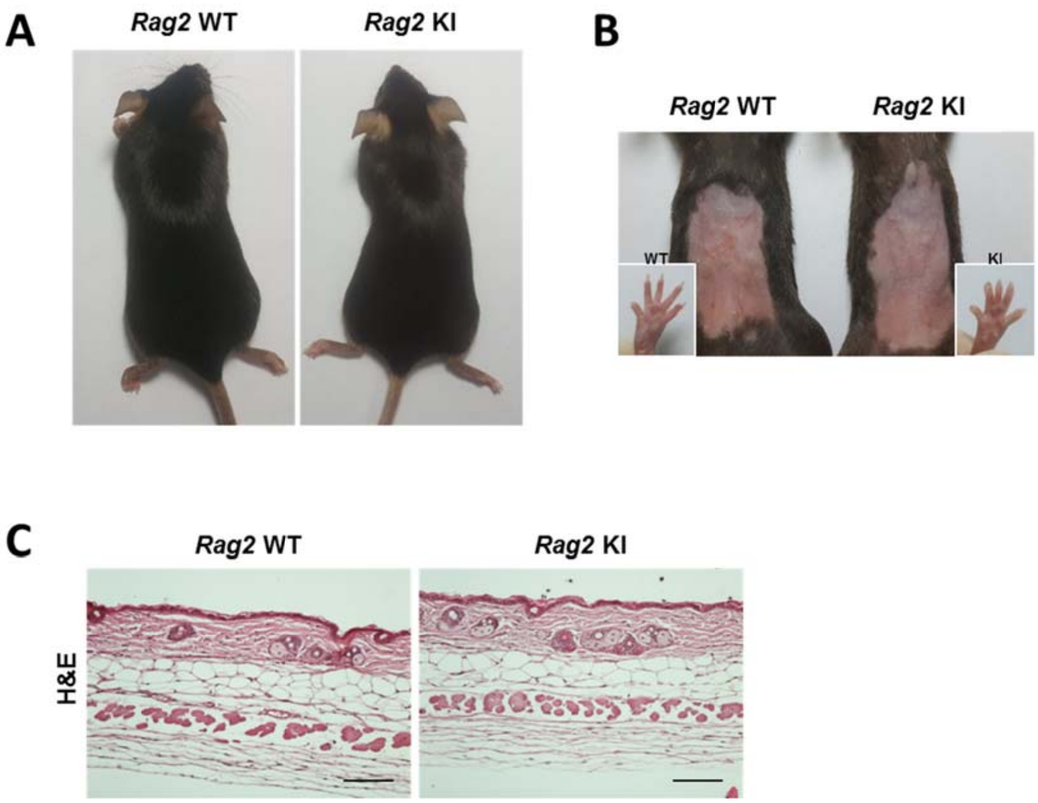

Supplementary Figure S1. Skin phenotypes of *Rag2* KI mice. (A-B) Gross morphology of 6-month-old female *Rag2* WT and KI mice. **(C)** Representative sections stained with hematoxylin and eosin (H&E) staining from dorsal skin of *Rag2* WT and KI mice. There were no significant differences of skin phenotypes between *Rag2* WT and KI mice. Original magnification, x200; Scale bar, 100 μm.

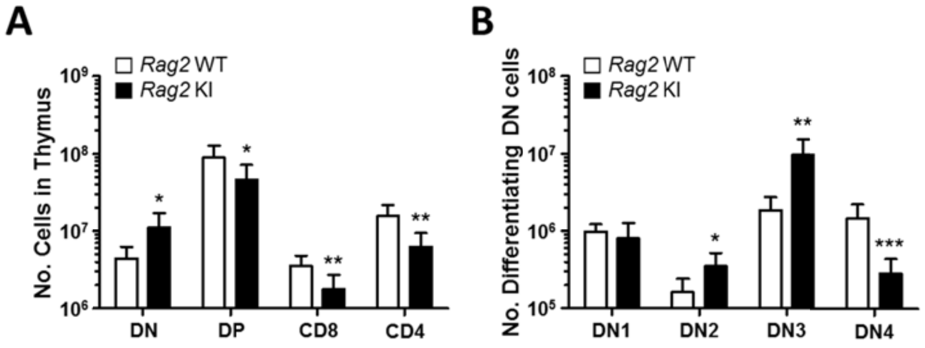

Supplementary Figure S2. Absolute numbers of T cells in the thymus of *Rag2* KI mice. Cells were isolated from the thymus of *Rag2* WT and KI mice. **(A)** Total number of $CD8^-CD4^-$ (DN), $CD8^+CD4^+$ (DP), $CD8^+$, and $CD4^+$ thymocytes. **(B)** Total numbers of $CD25^-CD44^+$ (DN1), $CD25^+CD44^+$ (DN2), $CD25^+CD44^-$ (DN3), and $CD25^-CD44^-$ (DN4) among $CD4^-CD8^-$ cells. Bar graphs show mean ± standard deviation (SEM). n=8 per group. *p<0.05, **p<0.01, ***p<0.001.

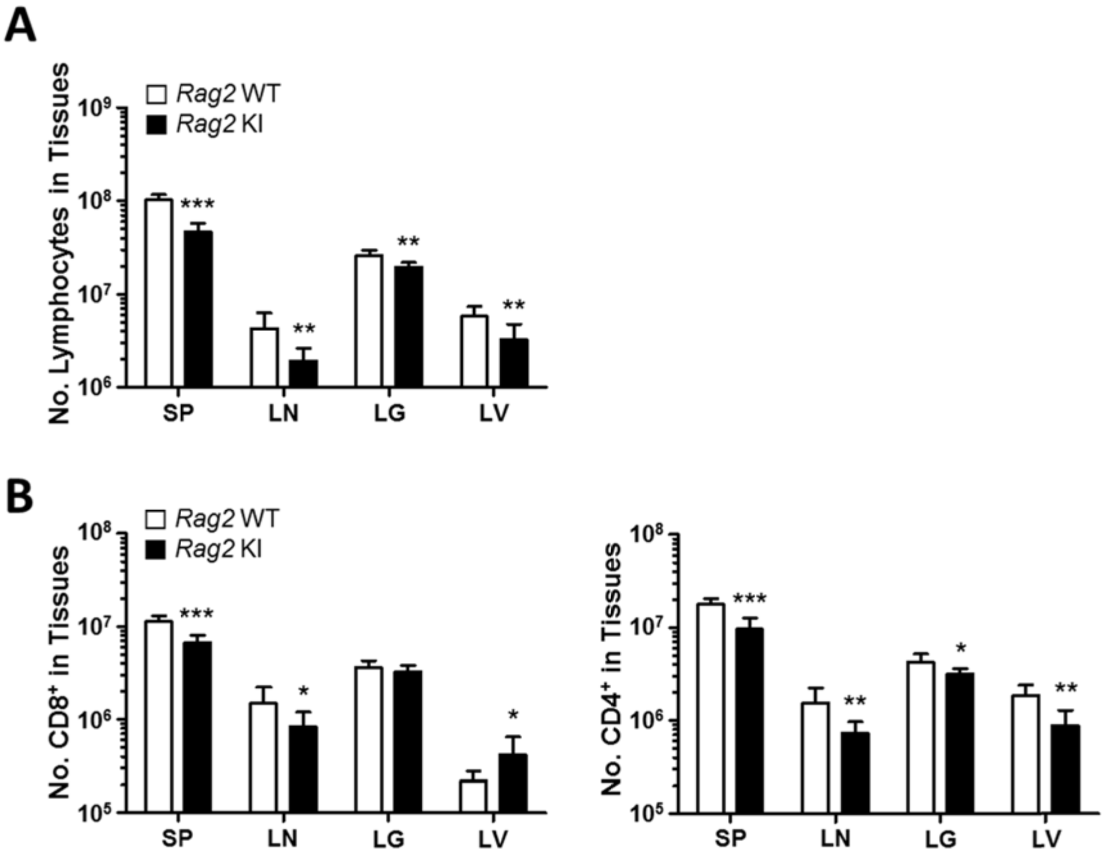

Supplementary Figure S3. Absolute numbers of lymphocytes and T cells in the lymphoid and non-lymphoid organs of *Rag2* KI mice. Cells were isolated from the spleen (SP), lymph node (LN), lungs (LG), and liver (LV) of *Rag2* WT and KI mice. **(A)** Total number of lymphocytes in the indicated tissues. **(B)** Left, total numbers of $CD8^+$ T cells in the indicated tissues. Right, total numbers of $CD4^+$ T cells in the indicated tissues. Bar graphs show mean ± SEM. n=8 per group. *p<0.05, **p<0.01, ***p<0.001.

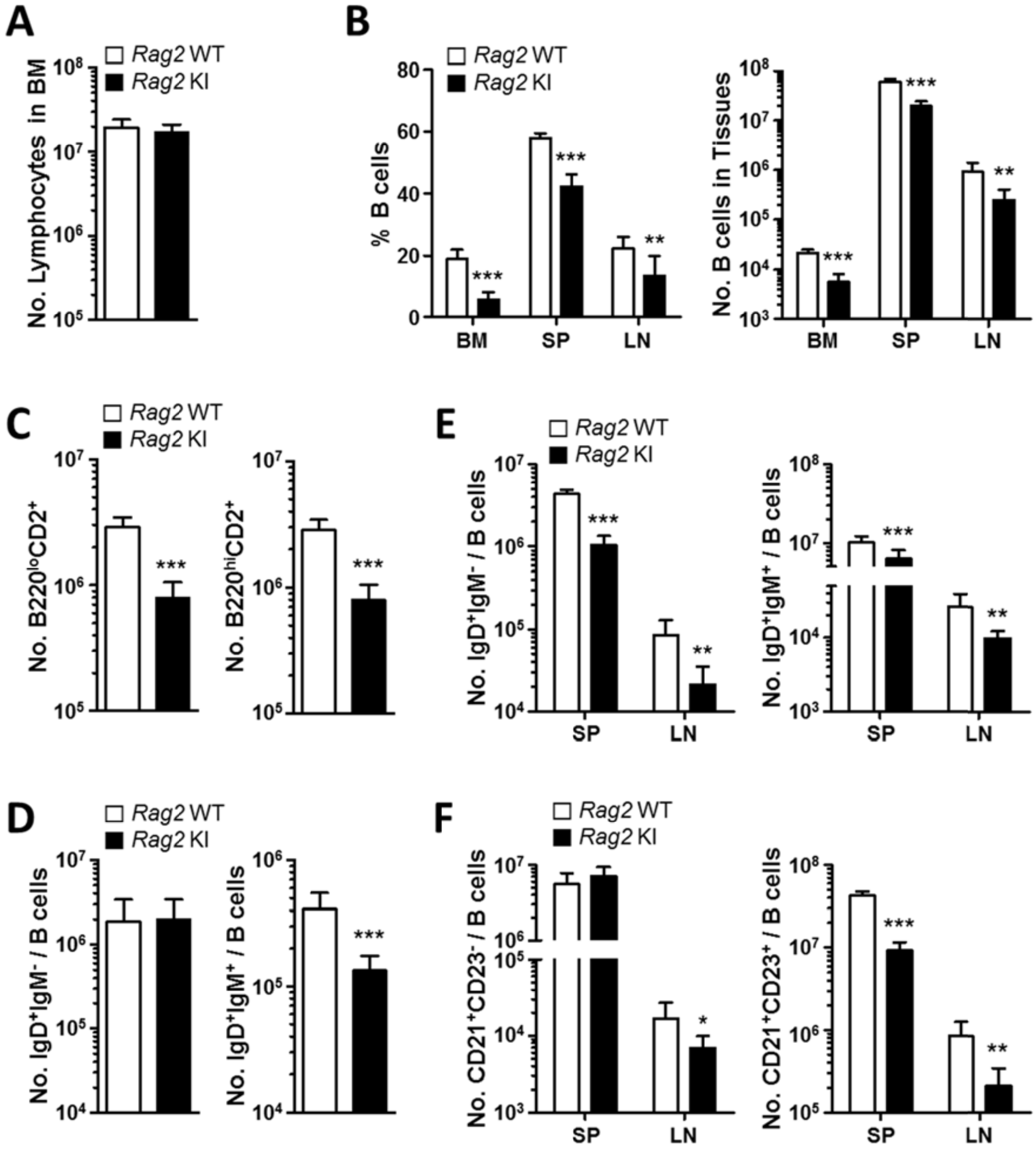

Supplementary Figure S4. Absolute numbers of B cells in the bone marrow, spleen, and lymph node of *Rag2* KI mice. Cells were isolated from the bone marrow (BM) **(A-D)**, spleen (SP), and lymph node (LN) (E and F) of *Rag2* WT and KI mice. **(A)** Total numbers of lymphocytes in the BM. **(B)** Left, frequency of B220^{+} B cells in the indicated tissues. Right, Total number of B220^{+} B cells in the indicated tissues. **(C)** The absolute numbers of B220^{lo}CD2^{+} or B220^{hi}CD2^{-} cells among B220^{+} cells in the BM were summarized in the bar graph. **(D)** The absolute numbers of IgD^{+}IgM^{-} and IgD^{+}IgM^{+} cells among B220^{+} cells in the BM are summarized in the bar graph. **(E)** The absolute numbers of IgD^{+}IgM^{-} or IgD^{+}IgM^{+} cells among B220^{+} cells in the SP and LN are summarized in the bar graph. **(F)** The absolute numbers of CD21^{+}CD23^{-} or CD21^{+}CD23^{+} cells among B220^{+} cells in the SP and LN are summarized in the bar graph. Bar graphs show mean ± SEM. n=8 per group. *$p<0.05$, **$p<0.01$, ***$p<0.001$.

Supplementary Table S1. Generation of F0 mice after CRISPR/Cas9-mediated gene targeting in C57BL/6JBomTac mice.

Target gene	Cas9 mRNA (ng/µl)	Total sgRNAs (ng/µl)	ssODN (ng/µl)	Scr7 (mM)	Injected embryos	Transferred embryos (%)	Live newborns	Founders* (%)	KI (%)
Rag2	20	200	200	1	168	140 (83.3)	27 (19.3)	23 (85.2)	1 (4.3)
	20	200	200	1	51	41 (80.4)	3 (7.3)	Not tested	-

*****Determined by polyacrylamide gel electrophoresis (PAGE)-based PCR genotyping.
